# Pollution Characteristics, Transport Pathways, and Potential Source Regions of PM_2.5_ and PM_10_ in Changchun City in 2018

**DOI:** 10.3390/ijerph17186585

**Published:** 2020-09-10

**Authors:** Fanhao Meng, Ju Wang, Tongnan Li, Chunsheng Fang

**Affiliations:** College of New Energy and Environment, Jilin University, Changchun 130012, China; mengfh18@mails.jlu.edu.cn (F.M.); litn2516@mails.jlu.edu.cn (T.L.); fangcs@jlu.edu.cn (C.F.)

**Keywords:** transport pathway, backward trajectory, cluster analysis, scene analysis, potential source contribution function

## Abstract

Air pollution has attracted increasing attention in recent years. Cluster analysis, scene analysis, and the potential source contribution function (PSCF), based on the backward trajectory model, were used to identify the transport pathways and potential source regions of PM_2.5_ and PM_10_ (particulate matter with an aerodynamic diameter of not more than 2.5 µm and 10 µm) in Changchun in 2018. In addition, the PSCF was slightly improved. The highest average monthly concentrations of PM_2.5_ and PM_10_ appeared in March and April, when they reached 53.9μg/m^3^ and 120.0 μg/m^3^, respectively. The main potential source regions of PM_2.5_ and PM_10_ were generally similar: western Jilin Province, northwestern Inner Mongolia, northeastern Liaoning Province, and the Yellow Sea region. The secondary potential source regions were southern Russia, central Mongolia, western Shandong Province, eastern Hebei Province, and eastern Jiangsu Province. The northwest and southwest directions were found to be the two pathways that mainly affect the air quality of Changchun City. Moreover, the northwestern pathway had a larger potential contribution source area than the southwestern pathway. The airflow in the southwest direction came from Liaoning Province, Shandong Province, and the Yellow Sea region. This mainly occurred in summer; its transmission distance was short; it had a relatively higher weight potential source contribution function (WPSCF) value; it can be regarded as a local source; and its representative pollutants were SO_2_ (sulfur dioxide), CO (carbon monoxide), and O_3_ (ozone). The northwestern pathway passed through Russia, Mongolia, and Inner Mongolia. The transmission distance of this pathway was longer; it had a relatively lower WPSCF value; it can be considered as a natural source to a certain extent; it mainly occurred in autumn and, especially, in winter; and the representative pollutants of this pathway were NO (nitric oxide), NOx (nitrogen oxide), PM_2.5_, and PM_10_.

## 1. Introduction

In recent decades, due to the rapid development of urbanization and the economy, China’s energy consumption level has continued to increase, and its air quality has dropped accordingly [1]. The air pollutants in China are complex mixtures of various substances [2]. To protect the environment and human health by preventing air pollution, China introduced the first and secondary concentration standards of CO, SO_2_, NO_2_, NO, NOx, O_3,_ PM_2.5_, and PM_10_ in the “Ambient Air Quality Standard” (GB3095-2012): For study areas located in the first-class ambient air functional area, the first concentration standard is applicable, and for those located in the second-class ambient air functional area, the secondary concentration standard is applicable. First-class ambient air functional areas are nature reserves, scenic spots, and other areas in need of special protection; second-class ambient air functional areas are residential areas, commercial mixed-traffic areas, cultural areas, industrial areas, and rural areas. The “Ambient Air Quality Standard” (GB3095-2012) is China’s national environmental quality standard, jointly issued by the Ministry of Environmental Protection and the General Administration of Quality Supervision, Inspection and Quarantine, which enforced it.

Air pollution is becoming increasingly serious, and this may have a severe impact on human life by increasing the incidence of disease and endangering human health [3,4]. In addition, epidemiological studies have shown that most air pollutants can cause respiratory tract infection and lung cancer and even shorten humans’ life span [5,6]. Among air pollutants, PM_2.5_ caused more than 1.3 million premature deaths in China in 2013 [7,8], and PM_2.5_ and PM_10_ were also the main research pollutant in many studies [9,10]. The main focus of research in recent years on air pollutants is mostly the variation in the mass concentration and composition of PM_2.5_. Wang et al. analyzed the annual, seasonal, and diurnal variations of PM_2.5_ concentrations, as well as the influence of the meteorological conditions in the five major cities of southern Hebei from 2013 to 2015 [11]. Zhang et al. analyzed the seasonal variations of PM_2.5_ and its major chemical compositions including water-soluble inorganic ions (WSII) and carbonaceous components in East China from 2014–2015 [12]. Moreover, the source apportionment of PM_2.5_ has also been the main focus of many studies, such as that of Peng et al., who investigated the characteristics and possible formation mechanisms of PM_2.5_ and its major chemical components in different seasons from October 2014 to July 2015 in Chongqing and analyzed the mass concentrations and chemical compositions [13]. Tunno et al. examined the source apportionment of indoor residential PM_2.5_ in the communities of Braddock and Clairtonthey in winter and summer. They identified 5-factor solutions for both seasons, which could explain 86–88% of the variability in the constituent concentrations [14].

Cluster analysis and scene analysis, based on the backward trajectory model, are effective methods for studying the characteristics of air pollutants’ transport pathways and diffusion [15,16,17]. Zhu et al. analyzed the pollutant transport path and potential sources during haze days in Hangzhou, Hefei, Nanjing, and Shanghai to characterize the haze pollution in the Yangtze River Delta [18]. Zhao et al. used correlation analysis, clustering analysis, and the backward trajectory model to understand the correlations of PM_2.5_ or ozone among the cities of the Sichuan Basin and the regional transportation of the two air pollutants in the city clusters of the basin [19]. Chen et al. used backward trajectory analysis, trajectory clustering techniques, potential source contribution function (PSCF), and CWT (concentration-weighted trajectory) to analyze the seasonal pattern of transport pathways and potential sources of fine particles in Chengdu based on the collection of annual hourly PM_2.5_ data [20]. In addition, PSCF has been widely used to identify the potential source region of atmospheric pollutants and particulate matter [21,22,23]. Qin et al. used the HYSPLIT model and its derived algorithms, which include the PSCF and CWT methods, at different scales to identify the major transport pathways and potential source areas of Artemisia pollen in Beijing [24]. Li et al. used cluster analyses, the PSCF method, and the CWT method, based on backward trajectories, to improve the understanding of the detailed transport pathways and potential sources of PM_2.5_ and PM_10_ in Beijing from June 2014 to May 2015 [25]. Xin et al. used cluster analysis, the PSCF method, and the CWT method to identify the major transport pathways of air masses and stimulate the main source areas to analyze the effects of the long-range transport pathways and potential sources of PM_10_ in Xining, China [26].

However, the focus of many related studies is often on the variation in the concentrations and transmission pathways of particulate matter in heavily polluted weather and haze weather. Less attention has been paid to the transport pathway of other air pollutants, and the results of PSCF have sometimes not been clear. In this paper, cluster analysis and scene analysis were used to identify the transport pathway and characteristics of the scenes of the annual, seasonal, and heating periods of CO, SO_2_, NO_2_, NO, NOx, O_3,_ PM_2.5_, and PM_10_ exceeded the standard in Changchun city in 2018. PSCF was used to identify and compare the potential source regions of PM_2.5_ and PM_10_. In addition, to make the result of PSCF clearer and more practical, we slightly improved the PSCF.

## 2. Principles and Methods

### 2.1. Air Pollution Data Source, Research Location and Measurement Instruments

**Figure 1:**
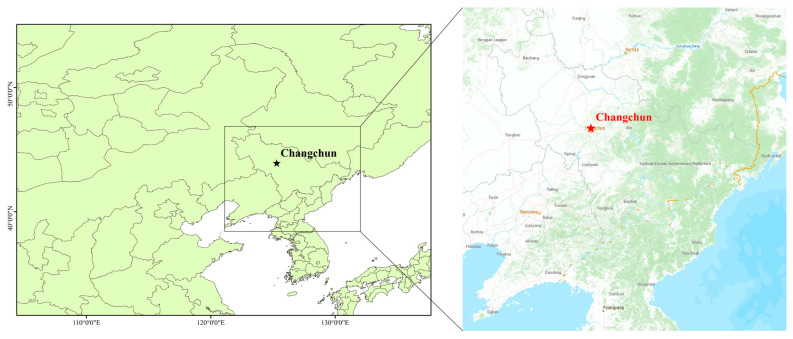
The research location of this study (the star represents the location of Changchun City).

Changchun is located in the northeastern region of China, the middle of the Northeast Plain and the central part of Jilin Province. The geographical location of Changchun is in the northern temperate zone of the middle latitude of the northern hemisphere. The meteorological type is the temperate continental humid climate type. Moreover, Changchun is the central city of the northeast Asian economic circle and a well-known old industrial base in China. It has a total population of approximately 7.513 million inhabitants and a total land area of 20,593.5 square kilometers (urban area of 7557 square kilometers) [27]. In this study, the hourly concentration data on PM_10_, PM_2.5_, SO_2_, NO_2_, NO, NOx, CO, and O_3_ in Changchun City in 2018 came from the Changchun Air Quality Monitoring Station. Its location (Longitude: 125.31° E, Latitude: 43.91° N) is shown in Figure 1.

The instruments and analytical methods used to measure the concentration of air pollutants were as follows: NO, NO_2_, and NOx were measured continuously using an NOx analyzer (Model 42i NO-NO_2_-NOx analyzer, Thermo Scientific, Waltham, MA, USA), which analyzed the ambient air using the principle of chemiluminescence at an interval of 1 min. SO_2_ was measured using an SO_2_ analyzer (Model 43i SO_2_ analyzer, Thermo Scientific, Waltham, MA, USA), which used pulsed fluorescence technology to measure SO_2_. CO was measured using a CO analyzer (Model 48i CO analyzer, Thermo Scientific, Waltham, MA, USA), which used gas filter correlation analysis technology. O_3_ was measured using an ultraviolet photometric ozone analyzer (Model 49i O_3_ analyzer, Thermo Scientific, Waltham, MA, USA), which was regularly calibrated using pure ozone and ozone-free air. PM_2.5_ and PM_10_ were measured using a SHARP Monitor (Model 5030i, Thermo Scientific, Waltham, MA, USA), which used the method of non-step measurement, combining the precision of the light scattering turbidity method with the accuracy of the β-ray absorption method. All the concentration data on PM_10_, PM_2.5_, SO_2_, NO_2_, NO, NOx, CO, and O_3_ used in this study were hourly monitoring values.

### 2.2. Cluster Analysis and Potential Source Contribution Function

In order to produce 3-day (72 h) backward air mass trajectories approaching Changchun at 500 m above ground level (AGL) over the whole year of 2018, the hybrid single-particle Lagrangian integrated trajectory (HYSPLIT) model of the National Oceanic and Atmospheric Administration (NOAA) was applied [28,29]. For each day, 24 trajectories reaching Changchun at 00:00–23:00 were computed. In addition, we also designed 14 scenes, which were as follows: (a) working day; (b) spring; (c) summer; (d) autumn; (e) winter(heating period); (f) non-heating period; (g) SO_2_ > 500 μg/m^3^; (h) NO > 100 μg/m^3^; (i) NO_2_ > 200 μg/m^3^; (j) NOx > 250 μg/m^3^; (k) O_3_ > 200 μg/m^3^; (l) CO > 10 mg/m^3^; (m) PM_10_ > 450 μg/m^3^; and (n) PM_2.5_ > 225 μg/m^3^. According to the conditions of different scenes, the trajectories that matched each scene were selected out of 8760 trajectories throughout the year. Then, cluster analysis was performed separately to identify the transport pathway of the air mass in different scenes. In this study, the number of clusters is set to 8.

For the purpose of identifying and comparing the potential source regions of PM_2.5_ and PM_10_, we implemented PSCF for PM_2.5_ and PM_10_ in this study. The limit concentration was selected from the secondary standard limit in the “Ambient Air Quality Standard” (GB3905-2012). For PM_2.5_, it is 75 μg/m^3^, and for PM_10_, it is 150 μg/m^3^. The PSCF grids cover a domain between 15 and 80° N and 55 and 150° E, with a 0.50° × 0.50° resolution.

In a meteorological sense, since Changchun City is located in northeast China, where the winter is cold and lasts longer, the heating period is also relatively longer, so we divided the four seasons into: 4.16–6.15 (spring), 6.16–8.31 (summer), 9.1–10.24 (autumn), and 10.25–4.15 (winter), which can also be regarded as the heating period, while the remaining periods can be regarded as non-heating periods. The limit of the standard of different pollutants in scenes (g)–(n) is the hourly average limit of the secondary standard in China’s “Ambient Air Quality Standards” (GB3905-2012).

Cluster analysis was used to classify and summarize trajectories to obtain the average trajectory that could represent numerous trajectories [30], and the stepwise cluster analysis method was used in this study [31]. The cluster analysis is expressed in Equation (1):

(1) $$D=\sqrt{\sum_{j=0}^{t}d_{j}^{2}}\;\mathrm{SPVAR}=\sum_{i=1}^{X}\sum_{j=0}^{t}D_{\mathrm{ij}}^{2}\;\mathrm{TSV}=\sum \mathrm{SPVAR}$$

In Equation (1), i is the number of the trajectories, j is the number of passing points, t is the movement time of the airflow, d_j_ is the distance between the jth point of the two trajectories, x is the number of trajectories in the cluster, D is the distance between the trajectories, so D_ij_ represents the distance between the jth passing point in the ith trajectory and the corresponding point on the average trajectory, SPVAR is the space variation of each group of trajectories, and TSV is the total space variation. The stepwise cluster analysis method could divide the adjacent points into the same category in a large number of statistical samples and then select the trajectories with a higher similarity for classification. As more categories were divided, the situation came closer to the real situation, and the error of the result became smaller.

The potential source contribution function (PSCF) is an algorithm that couples the backward trajectory model to the degree of air pollution at a specific site [32]. To calculate the PSCF, grid processing should first be performed on the area that the trajectories pass through. PSCF is expressed in Equation (2):

(2) $${\mathrm{PSCF}}_{\mathrm{ij}}=\frac{m_{\mathrm{ij}}}{n_{\mathrm{ij}}}$$

In Equation (2), n_ij_ is the sum of all trajectories’ transportation time when passing through the ijth grid, and m_ij_ is the sum of the transportation time of the trajectories whose concentration exceeds the limit in the ijth grid. The value of the PSCF reflects the possibility that the concentration of air pollutants exceeds the standard value when the air mass passes through the grid [33]. However, because the nature of PSCF is a conditional probability function, for some grids with a small number of trajectories, the calculated PSCF values will have great uncertainty. Thus, the weight function W_ij_ is introduced in order to reduce the error and make the result more accurate. Its value will depend on the relationship between the sum of the transportation time of all trajectories in a specific grid and the average residence time of each grid. In this study, W_ij_ is expressed in Equation (3):

(3) $$W_{\mathrm{ij}}=\{\begin{array}{c} 1.0\\ 0.7\\ 0.4\\ 0.2 \end{array}\begin{array}{c} \;\;\;\;\;\;\;n_{\mathrm{ij}}>90\\ \;\;\;\;\;\;\;\;45<n_{\mathrm{ij}}\leq 90\\ \;\;\;\;\;\;\;\;30<n_{\mathrm{ij}}\leq 45\\ \;\;\;\;\;\;n_{\mathrm{ij}}\leq 30 \end{array}$$

The PSCF_ij_ values were multiplied with W_ij_, and at last, the WPSCF is expressed as:

(4) $${\mathrm{WPSCF}}_{\mathrm{ij}}={\mathrm{PSCF}}_{\mathrm{ij}}\times W_{\mathrm{ij}}$$

However, combined with the research results from some studies [25,34,35], it could be found that even if the W_ij_ was introduced, the result of PSCF will still appear to show higher extreme values in some grids far away from the research area. This was due to the fact that the number of trajectories that passed through these grids was lower, so the total residence time was also lower. Therefore, we slightly changed the calculation method and defined the formula of PSCF as:

(5) $${\mathrm{PSCF}}_{\mathrm{ij}}=\frac{m_{\mathrm{ij}}}{n_{\mathrm{total}}}$$

In Equation (5), n_total_ is the sum of the residence time of all trajectories. A total of 8760 trajectories were used in this study, and the transmission time of each trajectory was 72 h. Thus, the value of n_total_ is 630,720. The new WPSCF value is recalculated, while the W_ij_ remains unchanged. However, the WPSCF values obtained by the improved method are relatively low, which is because the value of n_total_ is too large. This could make the result clearer and more practical, so we can distinguish the main and secondary potential source regions of PM_2.5_ and PM_10_ more easily by comparing the WPSCF values.

## 3. Results

### 3.1. Variation in Pollutant Concentrations

A comparison of the annual average value of the concentration of air pollutants in Changchun in 2018 and the annual average secondary concentration limit in China’s “Ambient Air Quality Standards” (GB3905-2012) is shown in Table 1. Because the research area is located in the second-class ambient air functional area, the secondary concentration standard is applicable. It can be seen that the annual average value of SO_2_ was clearly lower than the secondary concentration limit. However, the NOx was significantly higher than the secondary concentration limit, while the NO_2_ and PM_10_ were slightly higher than the secondary concentration limit, and the PM_2.5_ was slightly lower than the secondary concentration limit.

**Figure 2:**
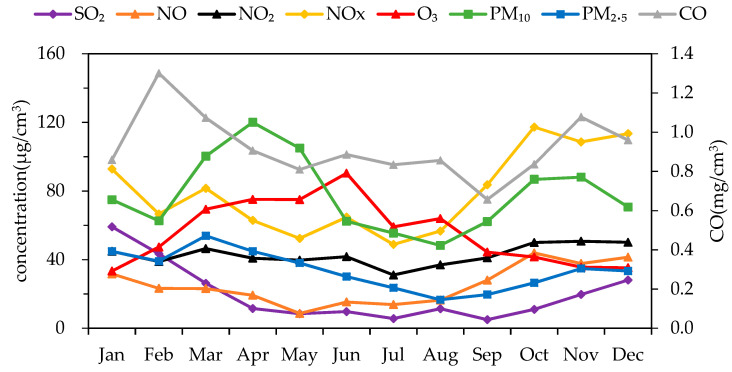
Monthly variation in the concentration of air pollutants in Changchun in 2018.

**Figure 3:**
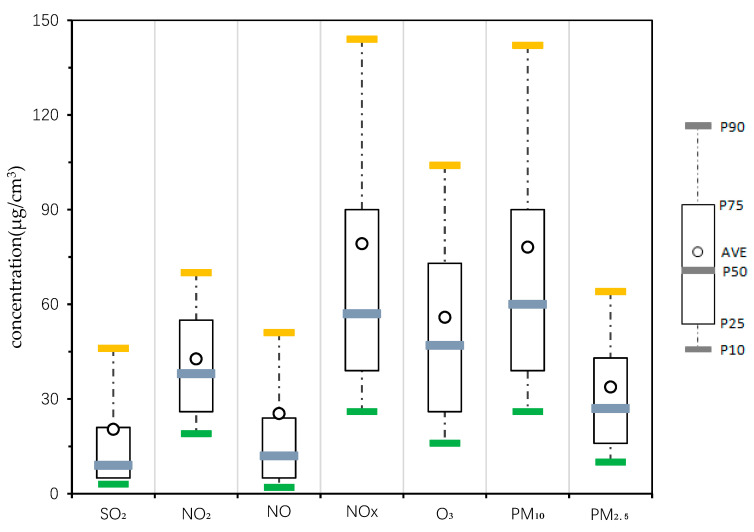
Hourly variation in the concentration of air pollutants in Changchun in 2018. (The circles represent the annual average concentration of air pollutants, and the horizontal lines represent the 90th, 75th, 50th, 25th, and 10th percentiles).

The monthly variation in the atmospheric pollutant concentration in Changchun City in 2018 is shown in Figure 2. It can be seen that the concentration variation in SO_2_, NO_2_, NOx, CO, PM_10_, and PM_2.5_ all showed the characteristics that the winter concentration was higher than that of other seasons, except for O_3_. O_3_ was obviously different from the other pollutants, whose concentration gradually increased from January to June and then decreased steadily until December. For PM_2.5_ and PM_10_, a slight decline occurred in February, when the mass concentration remained at a high level in January and then rose back to a higher concentration level in March. The highest average monthly concentrations of PM_2.5_ and PM_10_ also appeared in March and April, respectively (53.9 μg/m3 and 120.0 μg/m3, respectively). In addition, the hourly variation in the concentration of air pollutants in Changchun in 2018 is shown in Figure 3. It can be seen that, while the 90th percentile of NO was not very high, the annual average concentration of NO even exceeded the 75th percentile of NO’s hourly concentration. A few higher concentration values existed, and the SO_2_ also showed the same situation.

**Figure 4:**
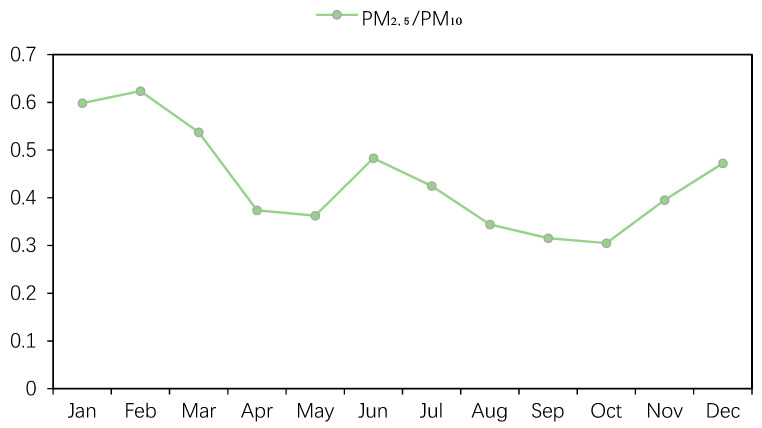
Variation in the PM_2.5_ and PM_10_ monthly average concentration ratios in Changchun in 2018.

In order to further elaborate the composition characteristics of atmospheric particulate matter, we also compared the PM_2.5_ mass concentration with the PM_10_ every month, as shown in Figure 4. It can be seen that the ratio of PM_2.5_/PM_10_ showed the characteristic of double peaks and double valleys, the ratio of the whole year fluctuated between 0.30–0.62, and the maximum value (0.62) and the minimum value (0.30) appeared in February and October, respectively. The ratio of PM_2.5_/PM_10_ was always higher than 0.5 during January to March, which means that PM_2.5_ accounted for more than half of the PM_10_ concentration at this time. The main component of atmospheric particulate matter was fine particles, more secondary particles were generated in the atmosphere, and the ratios of PM_2.5_/PM_10_ in June, July, and December were also still at a high level (above 0.4). September (0.31) and October (0.30) had relatively low ratios of PM_2.5_/PM_10_, which means that PM_2.5_ only accounted for a small proportion of PM_10_ at this time. Coarse particles were the main components of particulate matter pollution, and primary particles contributed more to the air pollution.

**Figure 5:**
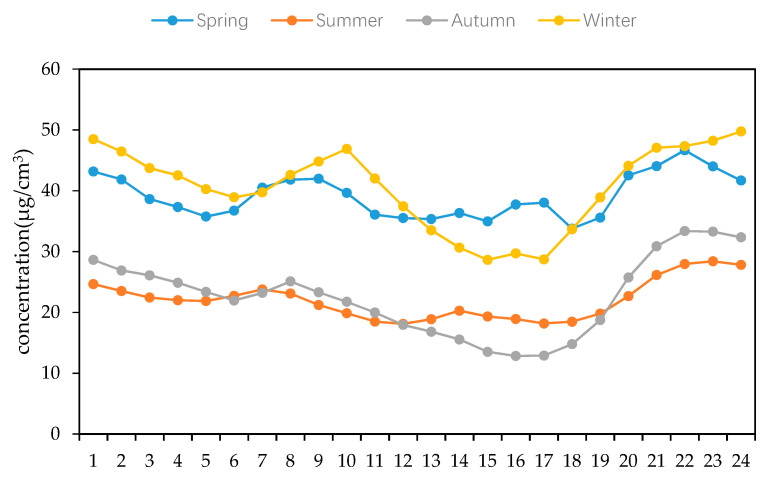
Variation in the hourly concentration of the PM_2.5_ concentration in the four seasons of Changchun City in 2018.

**Figure 6:**
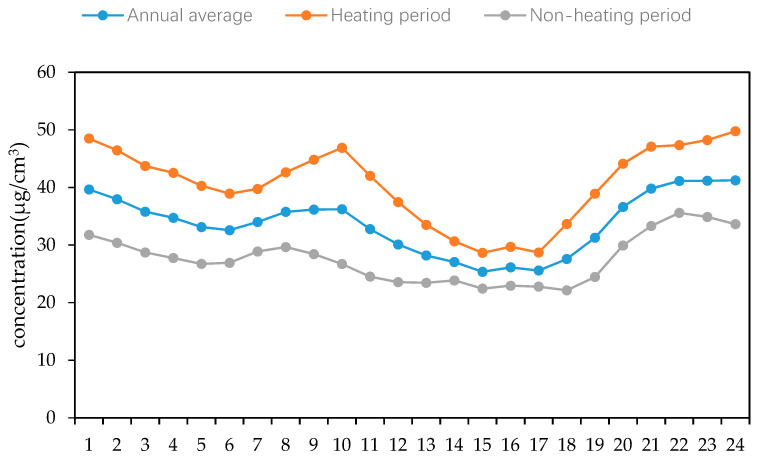
Variation in the PM_2.5_ concentration in the heating period and non-heating period in Changchun City in 2018.

In order to further study the performance of the PM_2.5_ concentration variation in different periods, according to changes at different stages during a 24 h day, the hourly average values of the PM_2.5_ mass concentration in the four seasons of 2018 in Changchun are calculated to show the changing characteristics of each day within each season. As shown in Figure 5, the concentration of PM_2.5_ basically followed the overall pattern of spring and winter, and was higher than that in summer and autumn, when the concentration of PM_2.5_ always remained at a low level. Similarly, in order to make the law of PM_2.5_ concentration variation more obvious, we used the same method to divide the PM_2.5_ mass concentration of Changchun City into two periods: a heating period and a non-heating period. Using these periods with the annual average value for the analysis shown in Figure 6, the characteristics of the PM_2.5_ concentration during the heating period > annual average > non-heating period were clearly shown, and a high ratio of PM_2.5_/PM_10_ was also shown to occur during this period. Combining this with the results shown in Figure 5 and Figure 6, the mass concentration of PM_2.5_ in Changchun City obviously showed a “double peak and double valley” distribution, with a high PM_2.5_ concentration at noon and during the night and a low concentration in the morning and evening.

### 3.2. Cluster Analysis

**Figure 7:**
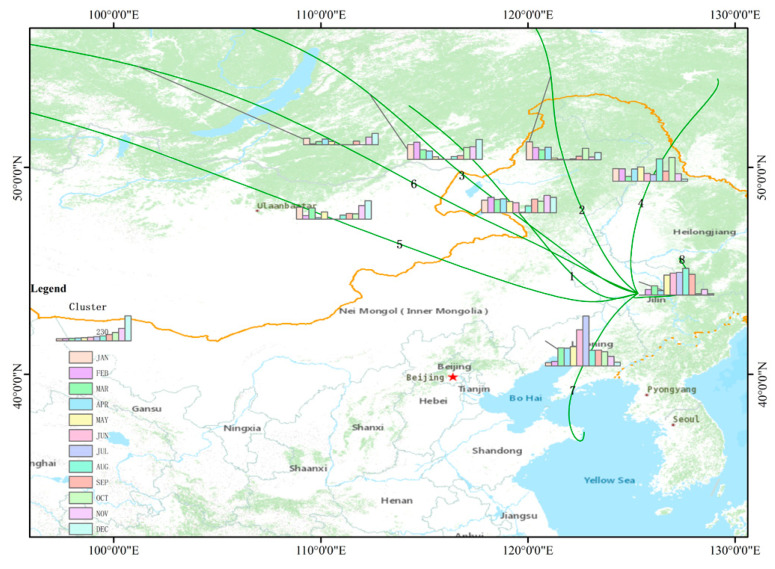
The average backward trajectories of Changchun City during the whole year of 2018. (the height of the columnar diagram indicates the quantity size of the average monthly trajectories).

As shown in Figure 7, a total of 8760 meteorological trajectories at a height of 500 m in Changchun during the whole year of 2018 were divided into 8 average trajectories according to the source and orientation of the airflow using the stepwise clustering analysis program. The point where the trajectories gathered is Changchun. The columnar diagram on the different average trajectories shows the quantity of trajectories occupied in different months. We numbered the clustering results as cluster 1–8. Specific information on the seasonal proportion of each cluster is shown in Table 2.

From the perspective of the whole year, most of the trajectories came from the northwest. Cluster 1 accounted for 15.17%, which was the second largest proportion of all the average trajectories, and the airflow of cluster 1 passed through southern Russia and eastern Inner Mongolia and finally turned slightly to the northeast to reach Changchun. The monthly distribution of cluster 1’s trajectories was relatively well-distributed, with only a proportion of trajectories in July and August being relatively lower. Clusters 2, 3, 5 and 6 also came from the northwest, especially cluster 3 (11.13%), which also passed through southern Russia and eastern Inner Mongolia to Changchun. Its trajectories mainly concentrated in January, February, September, November, and December, and the trajectories in May, June, July, and August only accounted for a very small proportion, so the contrast was very clear. Cluster 5 (8.69%) and cluster 6 (4.49%) both transported through southern Russia towards Mongolia, passing through the desert area of Mongolia and the Hunshandake Sand Lands of Inner Mongolia, finally reaching Changchun. Cluster 6 had the longest transmission distance of all the average trajectories. However, the proportion of trajectories was the lowest and the length of cluster 5 was also long, so this type of trajectories could belong to the long-distance transmission. Moreover, cluster 5 and cluster 6 both had almost no trajectories in June and July, and they were also relatively lower in May, which means that Changchun was susceptible to the airflow from the northwest direction in winter.

Cluster 7 (22.13%) had the largest proportion of all the trajectories. The first came from the northeast, which soon changed to the north–northwest direction, passing through northeastern Shandong and traversing central Liaoning Province. It finally reached Changchun from the southwest and passed through the Yellow Sea. Its quantity of trajectories was the highest in July, followed by June, which may indicate that Changchun was greatly affected by the southwest airflow in summer. In addition, the transmission distance of cluster 7 was the second shortest. Cluster 8 (14.95%) also belonged to short-distance transportation, and the vast majority of cluster 8 was transported over a short distance within the scope of Jilin Province, which means that cluster 7 and cluster 8 may be local sources to a certain extent.

### 3.3. Scene Analysis

**Figure 8:**
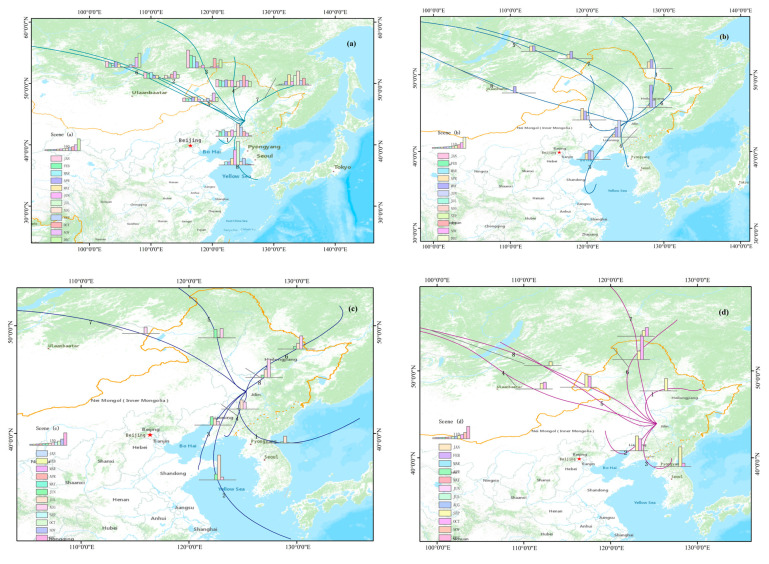

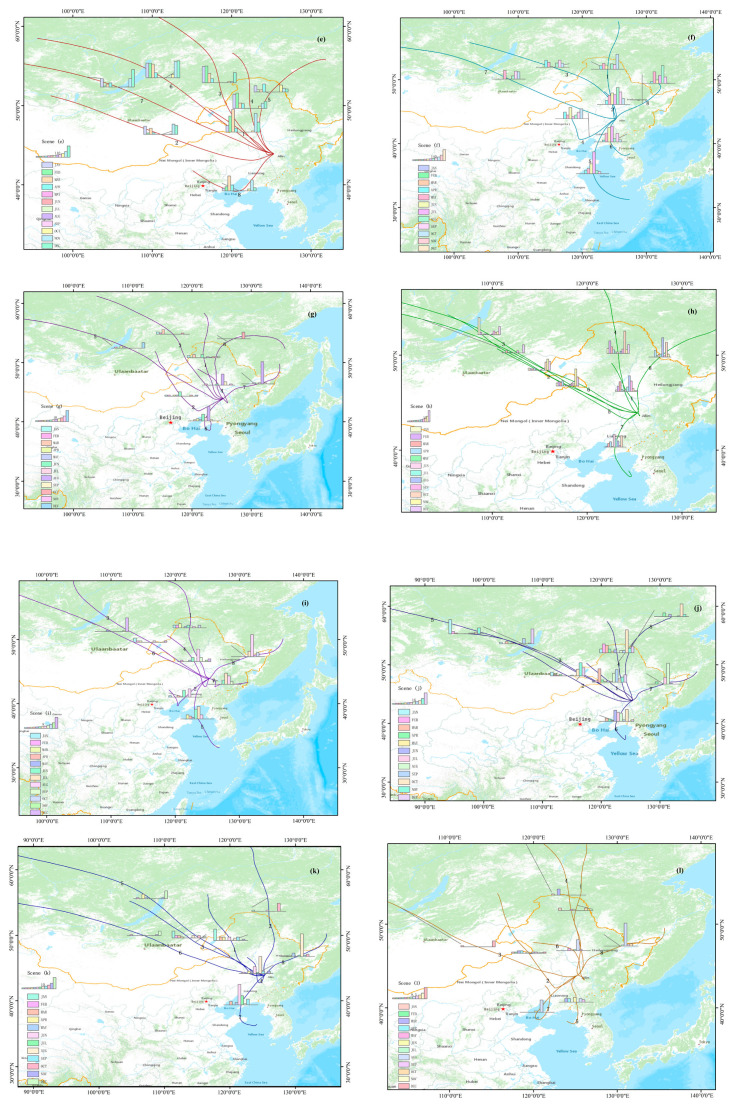

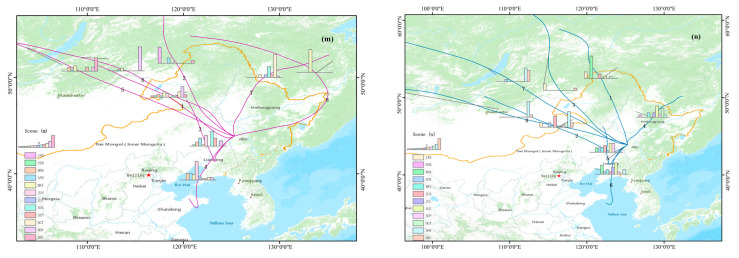
(**a**–**n**). Cluster analysis of the backward trajectories under specific scenes in Changchun in 2018: (**a**) working day; (**b**) spring; (**c**) summer; (**d**) autumn; (**e**) winter (heating period); (**f**) non-heating period; (**g**) SO_2_ > 500 μg/m^3^; (**h**) NO > 100 μg/m^3^; (**i**) NO_2_ > 200 μg/m^3^; (**j**) NO_x_ > 250 μg/m^3^; (**k**) O_3_ > 200 μg/m^3^; (**l**) CO > 10 mg/m^3^; (**m**) PM_10_ > 450 μg/m^3^; (**n**) PM_2.5_ > 225 μg/m^3^ (the height of the columnar diagram indicates the quantity size of the average monthly trajectories).

As shown in Figure 8a, the results of scene (a) were similar to the annual results shown in Section 3.2, both of which were mainly affected by the trajectories from the northwest. The subtle difference is in cluster 1, which originated in the west of Liaoning Province and passed through the Bohai Sea, reaching Changchun from the southwest. Like cluster 1, cluster 8 also had a higher proportion of trajectories in summer, which means that Changchun was susceptible to the airflow from the southwest in summer.

The scene of summer is shown in Figure 8c. The short-distance transportation represented by cluster 8 (18.94%) had the largest quantity of trajectories in summer, and the airflow mainly came from the west and southwest (clusters 1–4). These four average trajectories passed more or less through the sea surface, and the airflow from the sea surface was generally clean, which also improved the air quality in summer.

For the air pollutants in summer, Figure 8g showed the trajectories of the scene when SO_2_ > 500 μg/m^3^. Cluster 7 (20.91%), which came from the northeast, and cluster 6 (24.55%), which came from the southwest, had the highest proportion of trajectories in this scene. They both belonged to short-distance transportation near Jilin Province, which means that the SO_2_ concentration in Changchun was mainly affected by short-distance transportation in neighboring provinces and mainly in summer.

The situations of CO and O_3_ were also the same as that of SO_2_. Figure 8k shows the trajectories of the scene when O_3_ > 200 μg/m^3^. The frequency of the trajectories occurring in this scene was higher in June and August. Cluster 4 (18.29%) passed through Inner Mongolia and the northwestern Liaoning provinces, and cluster 7 (22.56%) came from the Yellow Sea, northeastern Shandong Province, and central Liaoning Province. Cluster 4 and cluster 7 were the top two average trajectories in this scene, and they were both significant in summer.

Figure 8l showed the trajectories of the scene when CO > 10 mg/m^3^. It can be seen that the direction of the airflow movement in this scene was extremely complicated. However, the frequency of the occurrence of the trajectories was extremely high in August. Cluster 8 (25.56%) came from southwestern Russia and passed through central Heilongjiang Province. It had the highest proportion of trajectories both in this scene and in August.

The scene of winter (heating period) is showed in Figure 8e. It can be seen clearly that all of the average trajectories, except that of cluster 5, came from the west and mainly concentrated in the northwest direction, which accounted for 82.95% of all trajectories. This means that Changchun was mainly affected by the airflow from the northwest in winter (heating period).

Figure 8d shows the scene of autumn. It can be seen that half of the clusters’ transmission directions changed significantly (cluster 1, cluster 2, cluster 3, and cluster 6), although the three clusters that came from the northwest did not change. The proportion of the quantity of the trajectories was relatively lower (cluster 4, cluster 5, and cluster 8 accounted for a total of 21.93%), indicating that the wind direction of the airflow changed many times during this period.

For the air pollutants in autumn and winter, Figure 8h shows the trajectories of the scene when NO > 100 μg/m^3^. The trajectories were mostly concentrated in the northwest. It can be seen that cluster 2, cluster 3, cluster 5, and cluster 6 all came from Russia, passing through the Baikal Lake and the Hunshandake Sand Lands of Inner Mongolia and then reaching Changchun. Cluster 1 (15.15%) and cluster 4 (19.80%) also came from the northwest and were the top two average trajectories in this scene. This means that the NO in Changchun was susceptible to the airflow from the northwest.

Figure 8j shows the trajectories of the scene when NOx > 250 μg/m^3^. It can be seen that the trajectories mostly came from the northwest, and the frequency of the occurrence of this scene was extremely high in October. Cluster 4 (21.43%) came from the northwest, and cluster 6 (15.71%) crossed the Yellow Sea and passed through Liaoning Province. Cluster 4 and cluster 6 both belong to short-distance transportation, which means that the NOx was mainly affected by the airflow of short-distance transportation, which was especially significant in autumn.

The scene when PM_10_ > 450 μg/m^3^ is shown in Figure 8m. It can be seen that the trajectories were mostly long-distance transportation in this scene. Except for cluster 3 and cluster 4, all of the average trajectories passed through southern or southeastern Russia and the Hunshandake Sand Lands in Inner Mongolia, which means that Changchun was susceptible to particulate matter pollution from Russia and Inner Mongolia. Moreover, it is worth mentioning that cluster 3 and cluster 4, which came from the southwest, also had a relatively higher proportion of the quantity of trajectories, they both belonged to short-distance transportation, and they may be the trajectories of local sources to a certain extent.

The results of scene(m) and scene(n) were nearly the same. Figure 8n shows the trajectories of the scene when PM_2.5_ > 225 μg/m^3^. The frequency of the occurrence of this scene was relatively higher in March and November. In particular, the trajectories of March had an extremely high level in cluster 1 (14.78%), and the trajectories of November had a relatively higher level in cluster 2 (18.26%), which came from the northwest. Moreover, cluster 5 and cluster 6, which came from the southwest, also had a higher proportion of trajectories and belonged to short-distance transportation. This means that the particulate matter pollution in Changchun was affected by the impact of Russia and Inner Mongolia. The airflow mostly belonged to long-distance transportation in the northwest direction, while the local source of particulate matter was mainly from the southwest direction, which belonged to short-distance transportation.

Figure 8f shows the trajectories of the scene of the non-heating period. Cluster 2 (19.68%), which had the highest proportion of trajectories, also had an extremely short transportation distance. The proportion of trajectories in the northwest direction, as the main direction of the contribution source during the heating period, was extremely low during the non-heating period, with only 15.48% (the sum of cluster 3 and cluster 7), which means that the main contribution source direction during the non-heating period was the south direction.

Figure 8b showed the scene of spring, which included the following: cluster 2 (16.05%), coming from the Hunshandake Sands Land in Inner Mongolia; cluster 4 (18.99%), coming from eastern Liaoning Province and passing through the Yellow Sea; and cluster 6 (20.49%), coming from central Heilongjiang Province. All of these belonged to short-distance transportation and had a relatively higher proportion of trajectories, meaning that Changchun was mainly affected by short-distance airflow in spring.

Figure 8i showed the scene when NO_2_ > 200 μg/m^3^. Cluster 5 (18.93%), coming from the southeast, cluster 8 (17.42%), coming from the northeast, and cluster 7 (14.39%), coming from the east, were the top three average trajectories in this scene, which means that the concentration of NO_2_ in Changchun was susceptible to the airflow from the east.

### 3.4. Potential Source Regions

**Figure 9:**
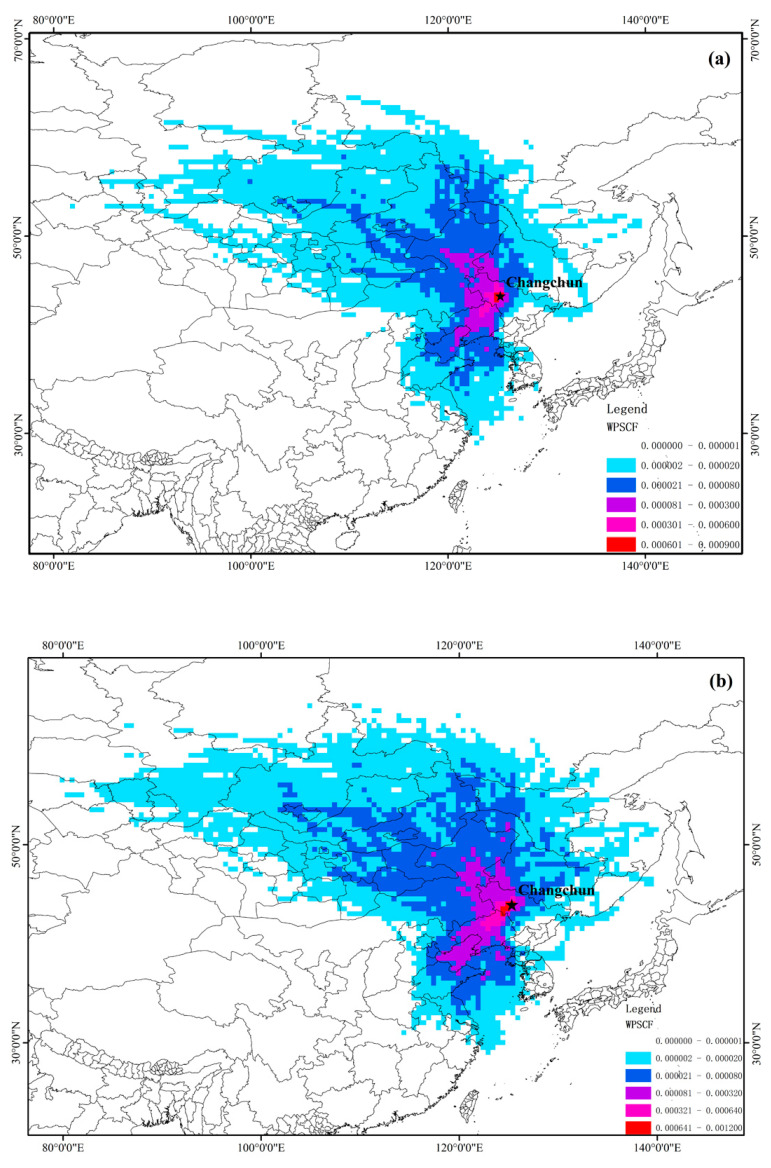
The weight potential source contribution function (WPSCF) value of (**a**) PM_2.5_ and (**b**) PM_10_ in Changchun in 2018. (The star represents the location of Changchun City).

The result of PSCF is shown in Figure 9. It can be seen that the regions of the high WPSCF values of PM_2.5_ and PM_10_ were all the western regions. In the southwest direction, the WPSCF values of PM_2.5_ and PM_10_ reached 0.0003–0.0006 at the juncture of southwestern Jilin Province and northwestern Liaoning Province. Moreover, we observed that the variation in the WPSCF values of the grid in the southwest was also relatively significant in Figure 9a,b, and the values of WPSCF were also relatively higher, while the WPSCF values were lower in the coastal regions of western Shandong, eastern Hebei Province, and eastern Jiangsu Province, reaching 0.00002–0.00008.

In the northwest direction, while the variation in the WPSCF values were slower than that in the southwest, the area of the potential contribution source regions of PM_2.5_ and PM_10_ in the northwest was significantly larger than that in the southwest. The high values of WPSCF in the northwest were mainly concentrated in northwestern Jilin Province, western Heilongjiang Province, and southwestern Inner Mongolia, reaching 0.00008–0.0006.

In addition, we can see that PM_10_ was more susceptible to the airflow from the northwest than PM_2.5_. The WPSCF values of PM_10_ reached 0.00002–0.00008 in almost the entire Donod Province and Sukhbaatar Province. However, the WPSCF values of PM_2.5_ were mostly only 0.000002–0.00002 in these regions. The same situation also existed in the southwest. The WPSCF values of PM_10_ reached 0.00008–0.0003 almost in the entire Yellow Sea, while the WPSCF values of PM_2.5_ were mostly 0.00002–0.00008 in this area.

## 4. Discussion

The results of this study showed that the concentration variation in PM_2.5_ generally has the following pattern: a high concentration at noon and during the night; a low concentration in the morning and evening; a low concentration in February; a high concentration in January and March; a higher concentration in winter than the other seasons; and the heating period > annual average > non-heating period. The possible causes for these phenomena are air pollutants discharged into the atmospheric environment by motor vehicles during the morning peak, which gradually accumulate to a peak value in the period near noon. However, the sunlight became stronger, and the air humidity decreased after noon, so the concentration of particulate matter was minimized around 17–19 h. The evening peak of travel was also at this time, and the height of the mixed layer decreased, so the concentration of particulate matter gradually accumulated to another highest value at around 23 h. The concentration of particulate matter slowly decreases with the cessation of human activities at night, then falls to a relatively low value in the morning. China’s annual Spring Festival was usually in February, when the factories and enterprises stopped work to rest, and the number of motor vehicles on the road was greatly reduced. After the end of the Spring Festival holiday in March, the factories and enterprises resumed production, which brought the pollutant concentration to a higher level.

The influence of heating was obvious. Frasca et al. studied the influence of airtight wood-fired appliances on indoor air quality and explained that a biomass-fueled heating system was a significant source of indoor pollution [36]. Winter in northern China was significantly cold, and the heating period in Changchun lasted for a long time, which caused a serious increase in the content of coal dust in the atmospheric particulate matter in winter [37]. Moreover, the meteorological diffusion condition in spring and winter was not conducive to a diffusion of pollutants, resulting in a higher PM_2.5_ concentration in winter. The concentration of fine particulate matter in the air pollutants was also significantly increased in the surrounding cities of Inner Mongolia, Heilongjiang Province, and Liaoning Province during the winter heating period. With the strong northwest winds passing through Russia, Mongolia, and Inner Mongolia, then reaching Changchun after long-distance transportation, coarse particulate matter are converted into a large number of fine particulate matter due to the physical loss, which greatly reduces the atmospheric environment quality in Changchun and causes serious fog and haze. This was also one of the main reasons for the frequent occurrence of air pollution in Changchun in winter [38]. In addition, the months with a PM_2.5_/PM_10_ ratio greater than 0.4 were all within the heating period, except July, which could further illustrate the impact of the heating period, producing a large amount of fine particulate matter [39]. In addition, although the annual average of PM_2.5_ was lower than the secondary concentration limit, it was extremely close to the limit value, and PM_10_ was slightly higher than the secondary concentration limit, meaning that the control of the concentration of particulate matter still cannot be ignored.

The monthly concentration of O_3_ and NO_2_ showed the opposite characteristics, which was because NO_2_ and O_3_ could be converted into each other by a chemical reaction. The high temperature and the strong ultraviolet radiation of sunlight in summer were the most important factors for the generation of O_3_ through photochemical reactions [40]. However, the ultraviolet radiation in winter is lower, and motor vehicle emissions and heating, coupled with the phenomenon of inversion, which occurs frequently in winter, also lead to high NO_2_ emissions. Moreover, the annual average of NO_2_ was slightly higher than the secondary concentration limit, but NOx was significantly higher than the secondary concentration limit, which may be related to the high annual average of NO. This means that we need to strengthen the control of the concentration of NOx.

From the perspective of the whole year, on the one hand, nearly half of the trajectories came from the northwest, and the columnar diagram of the northwest trajectories all showed a concave shape to a certain extent, meaning that the quantity of trajectories in autumn and winter was greater than that in spring and summer, which indicates that Changchun was susceptible to the airflow coming from the northwest during autumn and, especially, in winter. On the other hand, summer is mainly affected by the southwest airflow, and the columnar diagram of the southwest trajectories clearly showed a concave shape. In addition, the speed of the airflow movement could be determined according to the length of the trajectories. Long trajectories correspond to fast-moving air mass, while short trajectories correspond to slow-moving airflow. The airflow coming from the southwest passed through the Yellow Sea and belonged to short-distance transportation, indicating that Changchun may be affected by the sea breeze blowing from the Pacific Ocean, which was significant in July and August. Generally speaking, the concentration of air pollutants in summer was lower than that in winter, which may be related to the dilution effect of the clean air transported from the Yellow Sea.

From the perspective of air pollutants, on the one hand, the representative pollutants in summer were SO_2_, O_3_, and CO, and their trajectories were mainly from the southwest, which is consistent with the results for the scene of summer. However, even if the proportion of trajectories was very high, the average trajectories contained only a few trajectories, indicating that although there did exist a phenomenon of air pollutants exceeding the standard, the actual frequency of their occurrence was quite low. China’s coastal regions were vulnerable to the western Pacific subtropical pressure ridge in August, and typhoons were also more frequent at that time. Due to the double impact of the subtropical high and typhoons, a stable gas layer may have formed near the ground, which was conducive to the accumulation of atmospheric pollutants in a short period of time [41]. Moreover, due to the influence of the sea breeze, the clean air on the sea quickly dilutes the air mass with a high concentration of pollutants to reduce the frequency of the occurrence of a high pollutant concentration. On the other hand, the representative pollutants in winter and autumn were NO, NOx, PM_10_, and PM_2.5_, which were mainly affected by the airflow from the northwest, reflecting long-distance transportation, and the average trajectories of the air pollutants also passed through southern Russia and the Hunshandake Sand Lands in Inner Mongolia, meaning that Changchun was susceptible to pollution from Russia and Inner Mongolia. Moreover, the impact of the airflow coming from the southwest, which is mainly caused by short-distance transportation, cannot be ignored.

From the perspective of the potential source regions of PM_2.5_ and PM_10_, southwestern Jilin Province, northwestern Liaoning Province, and the Yellow Sea region were the main potential source regions of PM_2.5_ and PM_10_ of Changchun in the southwest direction. It is worth mentioning that the average trajectory with the highest proportion of trajectories also came from these regions. In addition, the airflow from the southwest mainly belonged to short-distance transportation, so it can be regarded as a local source contribution to a certain extent. Northwestern Jilin Province, western Heilongjiang Province, and southwestern Inner Mongolia were the main potential source regions of PM_2.5_ and PM_10_ of Changchun in the northwest direction. Compared with those of the southwest, the inland cities south of Liaoning Province have a relatively developed economy, and most of the airflow from the northwest came from southern Russia, Mongolia, and northwestern Inner Mongolia was transported over relatively longer distances. This is also related to the prevailing northwest wind in Changchun in winter. Particulate matter may undergo physical losses during long-distance transportation. The Hunshandake Sands Land in northwestern Inner Mongolia and the Gobi Desert regions in central Mongolia can also be regarded as natural sources of PM_2.5_ and PM_10_.

## 5. Conclusions

The concentration variations of SO_2_, NO_2_, NOx, CO, PM_10_, and PM_2.5_ all showed, to a certain extent, the characteristic of being higher in winter than in other seasons, with the exception of O_3_, and the concentration variation in PM_2.5_ generally has the following pattern: a high concentration at noon and during the night; a low concentration in the morning and evening; higher in winter than in other seasons; and the heating period > annual average > non-heating period. The highest monthly average concentrations of PM_2.5_ and PM_10_ (53.9 μg/m^3^ and 120.0 μg/m^3^) appeared in March and April, respectively. In addition, the ratio of PM_2.5_/PM_10_ was always higher than 0.4 from January to March and November to December, when the impact of the heating period was very obvious.

The northwest and southwest directions were the two pathways that mainly affected the air quality of Changchun City. On the one hand, Changchun was vulnerable to the airflow from the northwest, which came from Russia, Mongolia, and Inner Mongolia. The representative pollutants in this direction were NO, NOx, PM_10_, and PM_2.5_, which were especially significant in winter. The trajectories that came from northwest mainly belonged to long-distance transportation. On the other hand, Changchun was mainly affected by the airflow that came from southwestern Liaoning Province, Shandong Province, and the Yellow Sea region in summer. The representative pollutants in this direction were SO_2_, CO, and O_3_, and the trajectories from the southwest mostly belonged to short-distance transportation.

Generally speaking, the potential source regions of PM_2.5_ and PM_10_ in Changchun were substantially the same. Western Jilin Province, northwestern Inner Mongolia, northeastern Liaoning Province, and the Yellow Sea region can be regarded as the main potential source regions of PM_2.5_ and PM_10_ in Changchun. Southern Russia, central Mongolia, western Shandong Province, eastern Hebei Province, and eastern Jiangsu Province can be regarded as the secondary potential source regions of PM_2.5_ and PM_10_ in Changchun.

## Figures and Tables

**Table 1:** Comparison of the Annual Average Value of the Concentration of Air Pollutants in Changchun in 2018 and the Annual Average Secondary Concentration Limit in China’s “Ambient Air Quality Standards” (GB3905-2012).

	SO_2_	NOx	NO_2_	PM_10_	PM_2.5_
Annual average (μg/m^3^)	20.4	79.2	42.7	78.1	33.8
Annual average secondary concentration limit (μg/m^3^)	60	50	40	70	35

**Table 2:** Specific Information on Each Cluster in the Cluster Analysis.

Cluster	Direction of Each Cluster	Passing Area of Each Cluster	Percentage of Trajectories of Total Trajectories in Each Cluster	Quantity of Trajectories in Each Cluster	Percentage of Trajectories in Spring for Each Cluster	Percentage of Trajectories in Summer for Each Cluster	Percentage of Trajectories in Autumn for Each Cluster	Percentage of Trajectories in Winter for Each Cluster
1	NW–NE	Russia, Inner Mongolia, Jilin Province	15.17%	1329	15.05%	5.11%	17.68%	62.15%
2	NW	Russia, Inner Mongolia, Jilin Province	8.69%	762	2.89%	1.31%	18.50%	77.30%
3	NW	Russia, Inner Mongolia, Jilin Province	11.13%	975	2.62%	2.92%	15.08%	79.38%
4	NE–N	Russia, Heilongjiang Province, Jilin Province	14.50%	1271	16.13%	21.16%	24.78%	37.93%
5	NW–SW	Russia, Mongolia, Inner Mongolia, Jilin Province	8.90%	780	8.85%	5%	13.33%	72.82%
6	NW	Russia, Mongolia, Inner Mongolia, Jilin Province	4.49%	394	7.36%	0.25%	8.63%	87.36%
7	NE–NW–SW	Shandong Province, Yellow Sea, Liaoning Province, Jilin Province	22.13%	1939	26.71%	31.56%	14.44%	27.29%
8	NW–SW–W–SW	Heilongjiang Province, Jilin Province	14.95%	1310	29.85%	35.49%	15.88%	18.78%
